# Pressure injuries beyond acute care hospitals: a multicenter cohort study across rehabilitation settings

**DOI:** 10.3389/fresc.2026.1808329

**Published:** 2026-05-29

**Authors:** Luciana Bevilacqua, Lorenzo Brambilla, Guya Devalle, Letizia Oreni, Romina Puce, Davide Bertoletti, Luca Donati, Anna Pia Barberio, Cristina Messa

**Affiliations:** 1 IRCCS Fondazione Don Carlo Gnocchi, Milan, Italy; 2 School of Medicine and Surgery, University of Milano-Bicocca, Milan, Italy

**Keywords:** length of stay, older adults, patient safety, pressure injuries, rehabilitation

## Abstract

**Background:**

Pressure injuries (PIs) are widely recognized as indicators of patient safety and quality of care in acute hospitals, yet their burden in rehabilitation settings remains underexplored. Patients admitted to rehabilitation often present with high clinical complexity and functional dependency, potentially increasing PI-related risks.

**Methods:**

This multicenter retrospective cohort study was conducted across 15 inpatient rehabilitation facilities of the Fondazione Don Gnocchi network in Italy. All adult patients admitted for full-time rehabilitation between March 23 and April 23, 2024, were included, excluding those with stays shorter than 5 days. Data extracted from medical records included demographic and clinical characteristics, functional status (Barthel index), PI risk (Braden Scale), presence and characteristics of PIs at admission, incident PIs during hospitalization, healing outcomes, length of stay, and discharge destination. Prevalence and cumulative incidence were estimated, and cause-specific Cox regression models were applied to evaluate the association between PIs and time to routine discharge.

**Results:**

Among 1,013 patients, 175 had at least one PI at admission, corresponding to a prevalence of 17.3% [95% confidence interval (CI): 15.0%–19.8%]. Most lesions were stage 1–2 and primarily located at the sacrum and heels. Patients with PIs were older, more functionally dependent, and had higher rates of neurological comorbidities, dysphagia, incontinence, and medical device use. During rehabilitation, patients with PIs at admission showed a higher cumulative incidence of new PIs than those without (14.3% vs. 4.2%). Overall, 41.5% of lesions present at admission healed during hospitalization, with substantially lower healing rates for advanced-stage injuries. In multivariable analysis, the presence of PIs at admission was independently associated with prolonged time to routine discharge (adjusted hazard ratio 0.67, 95% CI: 0.54–0.83).

**Conclusions:**

Pressure injuries are highly prevalent at admission to rehabilitation and are associated with poorer clinical outcomes and increased resource utilization. These findings support the need for integrated preventive and management strategies spanning acute and rehabilitation care pathways.

## 1 Introduction

The prevalence of pressure injuries (PIs) in hospital settings remains high across Europe, with reported rates ranging from 4.6% to 27.2%, depending on the anatomical site involved (1). In Italian hospitals, prevalence rates recorded between 2010 and 2015 are comparable to European figures, ranging from 17% to 19.5% (2), thus representing a major challenge for healthcare systems. Although not all PIs are preventable, a substantial proportion could be avoided, given that the majority of hospital-acquired PIs (71.5%) are classified as stage 1 or 2 (3), and therefore may benefit from more effective preventive strategies.

Older adults are widely recognized as the population at greatest risk for PI development due to the presence of multiple predisposing factors, including comorbidities and functional impairments that contribute to frailty (4, 5). Specifically, reduced bed mobility, bowel incontinence, low body mass index, diabetes mellitus, peripheral vascular disease, advanced age, and the coexistence of urinary and fecal incontinence have been identified as key factors associated with an increased risk of PI development (5).

Pressure injuries have a substantial impact not only on patients' quality of life but also on length of stay and healthcare-related costs (6, 7). According to data from the Order of Nursing Professions of Bologna, approximately 4% of total expenditure of the Italian National Health Service (Servizio Sanitario Nazionale, SSN) is allocated to the management of complex wounds, with 15%–20% attributable to materials, 30%–35% to personnel, and nearly 50% to hospitalization (8). More recently, a Spanish study estimated the mean cost of treatment per patient to be approximately €1,400 for stage 1 PIs and up to €6,300 for stage 4 PIs (9). These findings underscore the need for effective preventive measures and targeted interventions to mitigate the clinical and economic burden of PIs.

In rehabilitation settings, the occurrence or persistence of PIs may be facilitated by specific conditions such as prolonged immobility, sensory impairments, altered peripheral perfusion, and the presence of comorbidities (10, 11). These factors coexist with well-established risk determinants reported in the literature, including sustained pressure, friction, moisture, malnutrition, and vascular dysfunction (12–14).

In addition to their role in the development of PIs, several clinical and care-related factors reflecting patient dependency and complexity, such as functional status, need for assistance with daily activities, and use of medical devices, may also affect time to discharge.

In Italy, a distinctive rehabilitation context is represented by the Fondazione Don Gnocchi (FDG), a non-profit organization managing 27 facilities, including two Scientific Institutes for Research, Hospitalization and Healthcare (IRCCSs), across nine regions. The FDG admits patients with high clinical and care complexity who require specialized rehabilitation programs—neurological, cardiological, or pulmonological—and who are transferred from both public and private acute-care hospitals.

Across all FDG facilities, including rehabilitation units and long-term care wards for older adults and individuals with disabilities, a prevalence survey on healthcare-associated infections was conducted for the first time in 2023–2024 (15). The survey aimed to quantify the magnitude of the problem across different healthcare and socio-healthcare settings and to identify potential areas for improvement in both preventive and clinical-care practices. In addition, the survey provided previously unpublished data on the clinical characteristics and complexity of patients admitted from acute-care hospitals, as well as on the specificity of rehabilitation interventions stratified by intensity of care, considering both clinical severity and nursing complexity.

The healthcare-associated infection study revealed significant differences across care settings; notably, intensive rehabilitation units were characterized by a higher proportion of complex patients and increased exposure to infections and antibiotic use (15).

Analysis of the collected data also yielded relevant findings regarding the prevalence of PIs in rehabilitation and socio-healthcare settings. Specifically, 17.6% of patients admitted to rehabilitation units presented with PIs of varying severity, while only 1.8% of patients developed PIs after admission.

Previous rehabilitation-hospital studies (16, 17) have shown that sacral PI development in older adults is associated with multiple comorbidities (e.g., dementia, Parkinson's disease, type 2 diabetes), longer length of stay, insurance status, and demographic and clinical factors such as age, sex, ethnicity, and malnutrition.

However, some of these determinants, such as insurance status, reflect healthcare system-related factors and are not directly applicable in the Italian context, where the National Health Service provides universal coverage ensuring access to care regardless of socioeconomic or insurance-related conditions. Moreover, a key limitation of prior research based on administrative data is the inability to clearly distinguish between PIs present at the time of admission and those acquired during the stay. This distinction is particularly relevant in rehabilitation settings, where patients often arrive from acute care with pre-existing lesions and remain exposed to ongoing risk factors such as immobility, dependence in activities of daily living, and comorbid conditions.

Building on this evidence, the present study does not aim to re-examine established risk factors for PI development. Instead, it extends previous work by providing a more granular analysis of pressure injury trajectories in rehabilitation patients, separately examining PIs present at admission, incident PIs occurring during rehabilitation, healing of pre-existing lesions, and their associations with clinically relevant outcomes, including time to routine discharge. This approach addresses an important gap in the literature and provides a clearer understanding of how both pre-existing and incident PIs contribute to the care burden in rehabilitation settings.

Finally, focusing on the healing of PIs present at admission and their impact on discharge timing may support the evaluation of the effectiveness of interventions for managing these lesions, with the aim of improving patients' quality of life.

## 2 Methods

### 2.1 Study design, setting, participants, and data collection

The aim of this multi-site retrospective cohort study was to estimate the prevalence of PIs at rehabilitation admission and to characterize patients with and without PIs. An additional aim was to examine their evolution and healing rate, assess their impact on length of stay, and estimate the cumulative incidence of PIs developing during the rehabilitation stay.

A total of 15 FDG facilities across Italy participated, including 6 in the north, 6 in the center, and 3 in the south. All patients admitted on a full-time basis (24 h/day) to the rehabilitation wards (neurological, cardiological, pulmonological, and orthopedic) between March 23, 2024 and April 23, 2024 were considered for inclusion in the study. Hospitalizations shorter than 5 days or of patients aged under 18 years were considered exclusion criteria.

Applying these inclusion and exclusion criteria, study participants were selected, and a total of 1,013 medical records were reviewed. The study involved collecting demographic and clinical characteristics of patients at admission to rehabilitation, including the primary diagnosis classified according to the International Classification of Diseases, Ninth Revision, Clinical Modification (ICD-9-CM) and the Diagnosis-Related Group (DRG) system, which classifies cases into Major Diagnostic Categories (MDCs), as well as the Barthel Index and the Braden Scale score as indicators of functional status. Additional information included type of diet, ability to feed independently, presence of urinary and/or fecal incontinence, and the use of medical devices such as urinary or vascular catheters.

For each patient, the presence or absence of PIs at the time of admission was recorded. When PIs were present, their number, anatomical location, and stage were documented. PIs were classified according to the National Pressure Ulcer Advisory Panel (NPUAP), European Pressure Ulcer Advisory Panel (EPUAP), and Pan Pacific Pressure Injury Alliance (PPPIA) guidelines, from stage 1, with intact skin and non-blanchable erythema, to stage 2, involving partial-thickness skin loss, stage 3, with full-thickness loss without exposure of bone, tendon, or muscle, and stage 4, with full-thickness loss exposing muscle, tendon, or bone. Unstageable injuries had bases obscured by slough or eschar, while suspected deep issue injuries (SDTIs) presented as intact or blistered skin with purple or maroon discoloration, indicative of deep tissue damage. All assessments were performed by trained healthcare professionals.

From the information collected in the medical records during hospitalization, data were extracted on pressure injuries (PIs) that developed after admission to rehabilitation, any acute events that occurred, the length of stay, and the type of discharge.

Finally, for each lesion, the occurrence of healing was recorded, along with the corresponding date. Data were collected using a REDCap database through the completion of study-specific case report forms (CRFs). Data entry was performed by 26 healthcare professionals who received dedicated training on the use of the application to minimize the likelihood of errors during data collection.

Details on all study variables, including source, operational definition, coding, reference categories, and whether variables were used for descriptive purposes only or included in multivariable analyses, are provided in the Supplementary Material (Supplementary Table S1).

### 2.2 Statistical analyses

Descriptive statistics were reported as percentages for categorical variables and as median values with interquartile ranges (IQRs) for continuous variables. Patients with and without pressure injuries at admission were compared using the Wilcoxon rank-sum test for continuous variables and the chi-square or Fisher's exact test for categorical variables, as appropriate.

Prevalence of PIs at admission and cumulative incidence of new PIs during hospitalization were calculated as percentages. Cumulative incidence was assessed separately for patients without PIs at admission (first occurrence) and for those with PIs at admission (additional lesions). Using the exact binomial method, 95% confidence intervals (CIs) were calculated.

For the analysis of healing of PIs present at admission, the statistical unit was the individual lesion. Since multiple lesions could occur in the same patient and therefore were not independent, a correction for intra-patient clustering was applied. Specifically, patient-level bootstrap resampling was used to account for the correlation among lesions within the same patient and to obtain robust confidence intervals for the estimates. The cumulative incidence function (CIF) of healing during hospitalization was estimated, treating death and discharge as competing events. This approach allows for accurate estimation of the cumulative probability of healing while properly accounting for both intra-patient dependence and competing risks.

To evaluate the effect of PIs on time to routine discharge, both univariable and multivariable cause-specific Cox proportional hazards models were used. Covariates included in the multivariable cause-specific Cox model were predefined *a priori* based on clinical relevance and their potential effect on time to routine discharge, in order to adjust for potential confounding of the association between PIs and this outcome. They were also selected to minimize multicollinearity, with variance inflation factors (VIF) used to verify that correlations among covariates were not problematic prior to model fitting. The proportional hazards assumption was assessed using the Schoenfeld residuals test. In addition, a sensitivity analysis was conducted using a Cox proportional hazards model with robust standard errors clustered by facility to account for potential within-facility correlation, with results reported in the Supplementary Material.

Death, transfer, and voluntary discharge were treated as competing events. The cause-specific hazard represents the instantaneous rate of routine discharge among patients still at risk, providing an appropriate estimate of the effect of PIs on discharge hazard.

Overall, approximately 10% of subjects had missing data for at least one covariate included in the Cox models. Given this relatively low proportion and under the assumption that missingness was random, no imputation methods were applied, and analyses were conducted using a complete-case approach, including only patients with complete data.

## 3 Results

The study included 1,013 subjects. Of these, 175 had at least one PI reported at admission to rehabilitation (Table 1), corresponding to a prevalence of 17.3% (95% CI: 15.0%–19.8%).

**Table 1:** Demographic and clinical characteristics of patients admitted to rehabilitation facilities according to the presence or absence of PIs at admission.

Characteristic	Total	No PIs	≥1 PIs	*P*
	(*N* = 1,013)	(*n* = 838)	(*n* = 175)	
Gender, *n* (%)				0.934
F	516 (50.9)	426 (50.8)	90 (51.4)	
M	497 (49.1)	412 (49.2)	85 (48.6)	
Age (years), median (IQR)	75 (64–82)	74 (64–81)	78 (64–85)	0.002
Number of PIs, *n* (%)				
0	838 (82.7)	838 (100.0)	–	
1	109 (10.8)	–	109 (62.3)	
2	47 (4.6)	–	47 (26.9)	
3+	19 (1.9)	–	19 (10.9)	
Patient origin, *n* (%)				0.076
Public hospital	654 (64.6)	527 (62.9)	127 (72.6)	
Accredited private hospital	154 (15.2)	136 (16.2)	18 (10.3)	
Home	101 (10.0)	90 (10.7)	11 (6.3)	
Residential facility	35 (3.5)	29 (3.5)	6 (3.4)	
Other	69 (6.8)	56 (6.7)	13 (7.4)	
MDC, *n* (%)				0.056
01	319 (31.5)	250 (29.8)	69 (39.4)	
08	400 (39.5)	335 (40.0)	65 (37.1)	
05	169 (16.7)	150 (17.9)	19 (10.9)	
04	68 (6.7)	55 (6.6)	13 (7.4)	
Other/Not available	57 (5.6)	48 (5.7)	9 (5.1)	
Comorbidities, *n* (%)				
Diabetes	245 (24.2)	197 (23.5)	48 (27.4)	0.286
Cardiovascular diseases	760 (75.0)	632 (75.4)	128 (73.1)	0.565
Chronic pulmonary diseases	162 (16.0)	128 (15.3)	34 (19.4)	0.175
Neurological diseases	128 (12.6)	91 (10.9)	37 (21.1)	<0.001
Muscle hypertonia, *n* (%)	59 (5.8)	41 (4.9)	18 (10.3)	0.011
Dysphagia, *n* (%)	180 (17.8)	123 (14.7)	57 (32.6)	<0.001
Modified-consistency diet, *n* (%)	222 (21.9)	163 (19.5)	59 (33.7)	<0.001
Feeding, *n* (%)				<0.001
Independent	617 (61.6)	553 (66.7)	64 (37.0)	
Partially assisted	283 (28.2)	215 (25.9)	68 (39.3)	
Fully assisted	102 (10.2)	61 (7.4)	41 (23.7)	
Braden score, median (IQR)	17 (15–20)	18 (16–20)	15 (13–17)	<0.001
Braden score, *n* (%)				<0.001
≥17—Minimal risk	549 (59.6)	504 (67.0)	45 (26.6)	
13–16—Low risk	317 (34.4)	224 (29.8)	93 (55.0)	
9–12—Moderate risk	47 (5.1)	22 (2.9)	25 (14.8)	
≤8—High risk	8 (0.9)	2 (0.3)	6 (3.6)	
Barthel Index, median (IQR)	45 (22–65)	50 (30–67)	20 (10–45)	<0.001
Barthel Index, *n* (%)				<0.001
≥61—Mild/no dependence	261 (27.0)	254 (31.9)	7 (4.1)	
41–60—Moderate dependence	258 (26.7)	220 (27.6)	38 (22.4)	
21–40—Severe dependence	211 (21.8)	174 (21.8)	37 (21.8)	
≤20—Total dependence	237 (24.5)	149 (18.7)	88 (51.8)	
Vascular access, *n* (%)				<0.001
None	731 (75.0)	641 (79.5)	90 (53.3)	
Peripheral	213 (21.8)	151 (18.7)	62 (36.7)	
Central	31 (3.2)	14 (1.7)	17 (10.1)	
Incontinence, *n* (%)				<0.001
None	687 (68.6)	607 (73.1)	80 (46.5)	
Urinary and fecal	169 (16.9)	114 (13.7)	55 (32.0)	
Urinary only	146 (14.6)	109 (13.1)	37 (21.5)	
Urinary catheter, *n* (%)	252 (24.9)	164 (19.6)	88 (50.3)	<0.001
Use of anti-decubitus devices, *n* (%)	305 (30.1)	175 (20.9)	130 (74.3)	<0.001
Prescription of mobilization, *n* (%)	879 (86.8)	711 (84.8)	168 (96.0)	<0.001
Restraints, *n* (%)	250 (24.7)	181 (21.6)	69 (39.4)	<0.001

IQR, interquartile range; PI, pressure injury; MDC, major diagnostic category.

1 = Diseases and disorders of the nervous system.

08 = Diseases and disorders of the musculoskeletal system and connective tissue.

05 = Diseases and disorders of the cardiovascular system.

4 = Diseases and disorders of the respiratory system.

The overall population, whose characteristics are reported in Table 1, had a median age of 75 years (IQR: 64–82 years), with 516 female subjects (50.9%). A total of 654 patients (64.6%) were transferred from public hospitals, 154 (15.2%) from accredited private hospitals, 101 (5.9%) from home, and 35 (3.5%) from residential facilities. Patients classified according to the MDC based on the referral diagnosis showed the following distribution: 319 subjects (31.5%) had diseases and disorders of the nervous system; 400 (39.5%) had diseases and disorders of the musculoskeletal system and connective tissue; 169 (16.7%) had diseases and disorders of the cardiovascular system; and 68 (6.7%) had diseases and disorders of the respiratory system. Comorbidities were mainly represented by cardiovascular diseases (75%), followed by diabetes mellitus (24.2%), chronic pulmonary diseases (16%), and neurological disorders (12.6%).

Motor dependency, assessed using the Barthel Index, was absent/mild in 27% of patients, moderate in 26.7%, severe in 21.8%, and total in 24.5%. The risk of developing pressure injuries, according to the Braden Scale, was classified as minimal in 59.6% of cases, low in 34.4%, moderate in 5.1%, and high in 0.9%. Regarding feeding, 61.6% of patients were autonomous, 28.2% required partial assistance, and 10.2% were fully dependent. Double incontinence was present in 16.9% of subjects, while 14.6% had urinary incontinence only. At admission, peripheral venous access was present in 21.8% of patients, urinary catheters in 24.9%, and central venous access in 3.2%.

Among the 175 patients with at least one PI at admission, most presented with a single lesion (62.3%), while 26.9% had two lesions, and 10.9% had three or more. The median age was significantly higher compared with patients without PIs [78 years (IQR: 64–85) vs. 74 years (IQR: 64–81), *p* = 0.002]. Compared with patients without PIs, those with PIs more frequently had neurological comorbidities (21.1% vs. 10.9%, *p* < 0.001), muscle hypertonia (10.3% vs. 4.9%, *p* = 0.011), dysphagia (32.6% vs. 14.7%, *p* < 0.001), and a higher rate of modified-consistency diet prescriptions (33.7% vs. 19.5%, *p* < 0.001).

Functionally and in terms of skin risk, patients with PIs had worse Braden Scale scores (median 15 vs. 18, *p* < 0.001), with a higher proportion at moderate-to-high risk, and significantly lower autonomy according to the Barthel Index (median 20 vs. 50, *p* < 0.001), with more than half classified as totally dependent. Patients with PIs at admission also showed a greater need for feeding assistance (*p* < 0.001), a higher prevalence of incontinence (*p* < 0.001), more frequent use of urinary catheters (50.3% vs. 19.6%, *p* < 0.001), and higher rates of peripheral or central vascular access (*p* < 0.001).

During hospitalization (Table 2), patients with at least one PI at admission had a higher cumulative incidence of new pressure injuries compared with those initially without PIs [14.3% (95% CI: 9.5%–20.4%) vs. 4.2% (95% CI: 2.9%–5.8%), *p* < 0.001]. Although differences in acute events—such as cardiovascular/respiratory events, nosocomial infections, falls, or mood alterations—were not statistically significant, patients with PIs had different discharge outcomes (*p* = 0.001), with a higher percentage of transfers to other facilities (20.6% vs. 11.0%). The median length of stay for all patients was 28 days (IQR: 19–42 days), and it was significantly longer in patients with PIs [median 35 days (IQR: 21–58 days) vs. 28 days (IQR: 19–40 days), *p* < 0.001].

**Table 2:** Clinical characteristics of patients during hospitalization.

Characteristic	Total	No PIs	≥1 PIs	P
	(*N* = 1,013)	(*n* = 838)	(*n* = 175)	
In-hospital acute events, *n* (%)				
Cardiovascular/respiratory event	40 (3.9)	29 (3.5)	11 (6.3)	0.081
Healthcare-acquired infection	41 (4.0)	33 (3.9)	8 (4.6)	0.699
Fall	27 (2.7)	20 (2.4)	7 (4.0)	0.228
Mood alteration	12 (1.2)	8 (1.0)	4 (2.3)	0.137
Onset of PI during hospitalization, *n* (%)	60 (5.9)	35 (4.2)	25 (14.3)	<0.001
Type of discharge, *n* (%)				0.001
Routine	862 (85.1)	730 (87.1)	132 (75.4)	
Transfer	128 (12.6)	92 (11.0)	36 (20.6)	
Death	11 (1.1)	8 (1.0)	3 (1.7)	
Voluntary	12 (1.2)	8 (1.0)	4 (2.3)	
Length of stay (days), median (IQR)	28 (19–42)	28 (19–40)	35 (21–58)	<0.001

PI, pressure injury.

**Figure 1:**
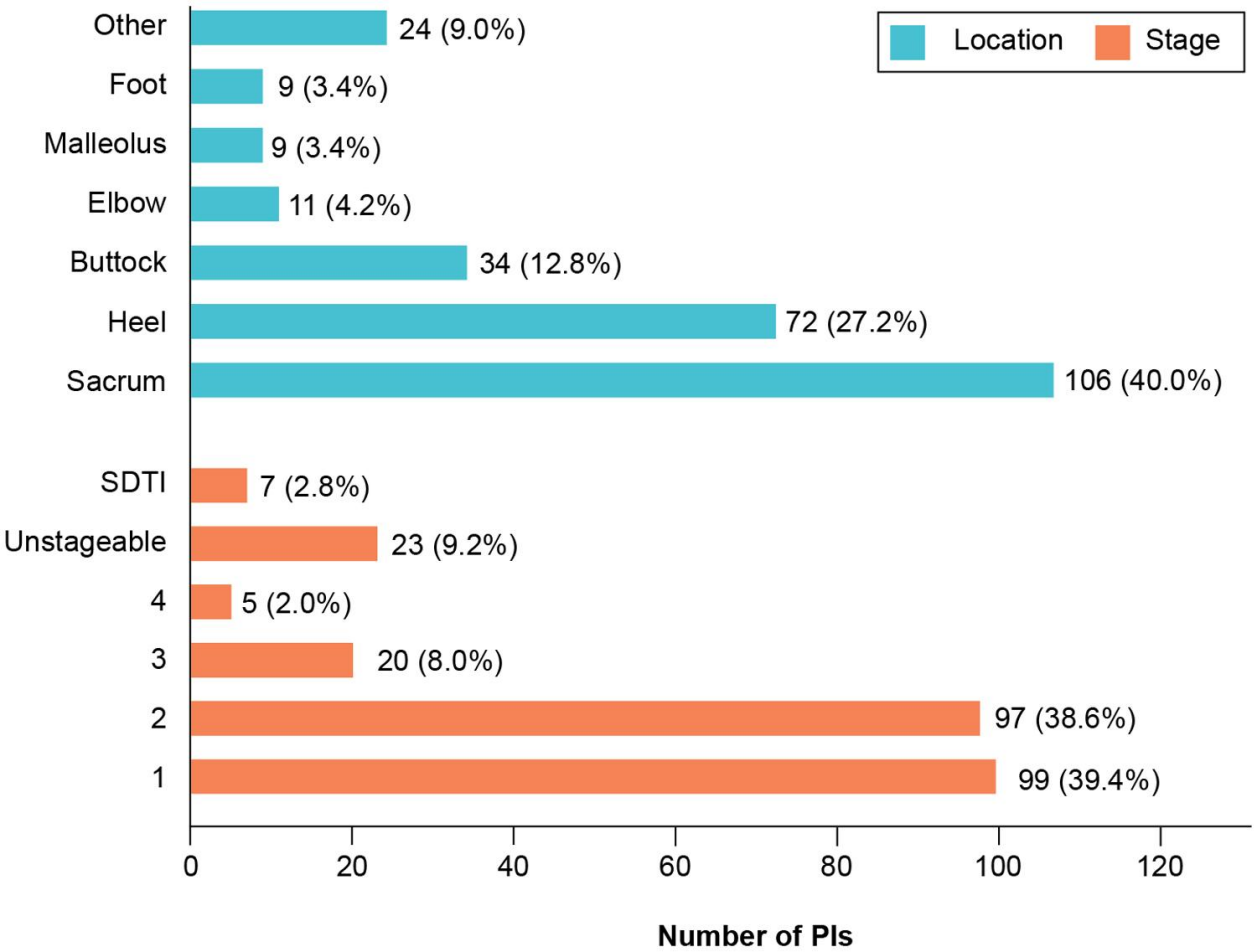
Anatomical distribution and staging of PIs present at admission.

The 175 patients with PIs at admission had a total of 265 lesions, predominantly stage 1 and 2 (39.4% and 38.6%, respectively). The most frequent anatomical locations were the sacrum (40.0%), followed by the heel (27.2%) and the buttocks (12.8%) (Figure 1). During hospitalization, 110 of these lesions (41.5%) healed.

**Figure 2:**
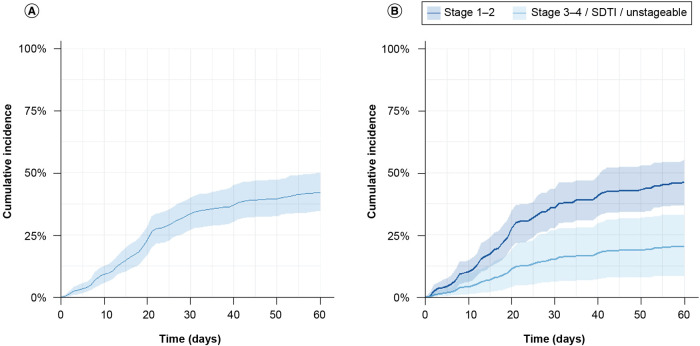
Cumulative incidence of healing for PIs present at admission. **(A)** Overall. **(B)** By lesion stage.

Within 30 days of admission, the cumulative incidence of healing for PIs present at admission was 33.4% (95% CI: 27.0%–39.9%) overall (Figure 2A). For stage 1–2 lesions, the estimated cumulative incidence was 36.0% (95% CI: 27.6%–43.5%), compared with 15.2% (95% CI: 5.9%–26.7%) for stage 3–4, SDTI, or unstageable lesions (Figure 2B).

In the multivariable analysis of time to routine discharge (Table 3), patients with at least one PI at admission had a lower hazard [adjusted hazard ratio (aHR) 0.67 (95% CI: 0.54–0.83), *p* < 0.001], indicating an extended time to routine discharge. Similarly, a prolonged time to discharge was observed in patients with a Braden score <17 [aHR 0.71 (95% CI: 0.60–0.84), *p* < 0.001], Barthel Index ≤ 40 [aHR 0.82 (95% CI: 0.68–0.98), *p* = 0.032], total feeding assistance [aHR 0.63 (95% CI: 0.44–0.92), *p* = 0.016], and the presence of a urinary catheter [aHR 0.77 (95% CI: 0.63–0.95), *p* = 0.016]. Compared with patients with cardiovascular diseases (MDC 05), those with diseases of the nervous system [MDC 01; aHR 0.22 (95% CI: 0.17–0.28), *p* < 0.001], musculoskeletal system and connective tissue disorders [MDC 08; aHR 0.40 (95% CI: 0.31–0.51), *p* < 0.001], and respiratory system disorders [MDC 04; aHR 0.47 (95% CI: 0.33–0.67), *p* < 0.001] also had an extended time to routine discharge.

**Table 3:** Univariate and multivariable cause-specific Cox regression analysis of factors associated with time to routine discharge.

Characteristic	HR	95% CI	P	aHR	95% CI	*P*
At least 1 PI at admission vs. none	0.57	0.47–0.68	<0.001	0.67	0.54–0.83	<0.001
Female vs. male	0.95	0.83–1.08	0.406	0.99	0.85–1.15	0.866
Age > 80 years vs. ≤80 years	1.06	0.92–1.22	0.442	1.09	0.93–1.29	0.279
MDC						
01 vs. 05	0.16	0.13–0.20	<0.001	0.22	0.17–0.28	<0.001
08 vs. 05	0.34	0.28–0.42	<0.001	0.40	0.31–0.51	<0.001
04 vs. 05	0.40	0.29–0.55	<0.001	0.47	0.33–0.67	<0.001
Other/Not Available vs. 05	0.21	0.15–0.29	<0.001	0.27	0.19–0.39	<0.001
Barthel index ≤40 vs. >40	0.45	0.39–0.52	<0.001	0.82	0.68–0.98	0.032
Braden score <17 vs. ≥17	0.49	0.42–0.57	<0.001	0.71	0.60–0.86	<0.001
Fully assisted feeding vs. partially assisted/independent feeding	0.29	0.22–0.39	<0.001	0.63	0.44–0.92	0.016
Presence of urinary catheter vs. no catheter	0.46	0.39–0.55	<0.001	0.77	0.63–0.95	0.016

aHR, adjusted hazard ratio; CI, confidence interval; HR, hazard ratio; PI, pressure injury; MDC, major diagnostic category.

01 = Diseases and disorders of the nervous system.

08 = Diseases and disorders of the musculoskeletal system and connective tissue.

05 = Diseases and disorders of the cardiovascular system.

04 = Diseases and disorders of the respiratory system.

The proportional hazards assumption was verified using the Schoenfeld residuals test, and no significant violation was detected (global *p* = 0.162), supporting the validity of the Cox model. In the sensitivity analysis accounting for clustering by facility, the main findings were generally confirmed. Notably, the association between LdP at admission and extended time to routine discharge remained statistically significant in the multivariable model, whereas a Barthel Index ≤ 40 and the presence of a urinary catheter were no longer significant (Supplementary Table S2).

## 4 Discussion

PIs continue to represent a major concern in healthcare, serving both as a marker of patient safety and as an indicator of the quality of nursing care. Our study confirms that PIs are not confined to acute hospital settings but are also highly prevalent in rehabilitation contexts. With 17.3% of patients presenting with at least one PI at admission, the problem is substantial, particularly considering that the majority of these lesions were stage 1–2, which are often preventable with timely and adequate interventions (1, 2). This finding underscores that PIs are a continuity-of-care issue, bridging acute and rehabilitation settings.

Patients with PIs at admission in our cohort were older, more functionally dependent, and exhibited more comorbidities, particularly neurological disorders, dysphagia, and immobility. These characteristics align with prior evidence highlighting that frail and neurologically impaired populations are at elevated risk for PIs (3, 4). Furthermore, the presence of urinary catheters, vascular access devices, and partial or full dependence in feeding were strongly associated with PIs, suggesting that device-related pressure and limited autonomy contribute significantly to skin breakdown. These findings emphasize the importance of comprehensive risk assessment at the time of admission, considering not only mobility but also medical devices, nutritional status, and comorbid conditions (5, 11).

Patients admitted to inpatient rehabilitation who cannot continue care in outpatient or day-care settings represent the most vulnerable population in our country, often with multiple comorbidities and potential social issues. In our study, approximately 83% of patients with PIs at admission were transferred from acute care hospitals, mostly public, and nearly half presented with vascular access (46.8%) and one-third with dysphagia (32.6%), reflecting the need for ongoing therapeutic interventions and suboptimal clinical stability.

Since PIs can affect patient management and the timely progression of rehabilitation, protective and preventive measures are implemented in our facilities to manage existing ulcers and reduce the risk of new lesions. These measures include the use of anti-decubitus mattresses, applied immediately in 74.3% of patients with PIs at admission and in 20.9% of those without, and mandatory mobilization, prescribed and carried out in 96% of patients with PIs at admission and in 84.8% of those without.

It is important to distinguish between the prevalence of PIs at admission and the incidence of new lesions developing during the rehabilitation stay, because prevalence at admission reflects patient complexity and prior care, while incidence during hospitalization is primarily influenced by preventive and monitoring measures.

Despite these interventions, 5.9% of patients developed new PIs. Patients with pre-existing PIs had a higher cumulative incidence of new lesions compared with those without PIs at admission (14.3% vs. 4.2%), experienced longer hospital stays, and were more frequently transferred to other facilities, highlighting their increased vulnerability and care needs. This shows the clinical and organizational burden of PIs, affecting resource allocation, staffing needs, and overall healthcare costs (7, 18). Healing rates were suboptimal, particularly for advanced-stage lesions, reflecting the complexity of these patients and the limitations of rehabilitation units in accelerating tissue repair for severe injuries (10, 11).

Nutritional status is a critical, yet often under-assessed, determinant of PI development and healing. In our cohort, the lack of systematic nutritional assessment, even during any previous acute hospitalization, hindered a thorough analysis of its role, mirroring findings in other rehabilitation settings (12, 13). Malnutrition can delay wound healing, increase susceptibility to skin breakdown, and contribute to prolonged rehabilitation stays. These results highlight the need for integrated, multidisciplinary strategies that include early nutritional screening, individualized dietary interventions, and careful monitoring throughout the rehabilitation process. The assessment of a patient's nutritional status must become a clinical parameter recorded in the medical documentation, with the same importance as other parameters (14).

The presence of PIs at admission, particularly among patients transferred from acute care hospitals who represent the majority of our cohort, suggests that these lesions may have developed during prior hospital care. This underscores the need for integrated care pathways. Improved communication between hospitals and rehabilitation centers regarding PI risk, preventive measures applied, and wound status at discharge could facilitate earlier interventions and reduce lesion progression. Moreover, structured prevention programs—such as scheduled repositioning, use of pressure-relieving devices, and patient and caregiver education—should be systematically implemented and continuously evaluated for effectiveness (19, 20).

Finally, our findings reinforce the broader implications of PIs on rehabilitation outcomes. Beyond clinical healing, the presence of PIs affects the ability to fully engage in rehabilitation programs, compromises functional recovery, and may influence psychological well-being. Addressing PIs comprehensively is therefore essential not only for wound management but also for optimizing rehabilitation efficacy, improving patient satisfaction, and reducing overall healthcare expenditure (21).

Despite these findings, certain limitations of our study should be considered when interpreting the results. All rehabilitation centers involved in this study are part of the FDG network. Given the organizational differences among rehabilitation facilities across Italy and the variability in regional healthcare regulations, generalization of our findings should be approached with caution. However, the results are consistent with those reported in other Italian and European studies (1, 2). Standardized monitoring tools were not routinely used in our cohort, which may have affected the reliability of PI healing assessment (22–24).

Moreover, the lack of systematic assessment of nutritional parameters limited the possibility to analyze the relationship between nutritional status and the incidence or progression of PIs. Future studies should incorporate standardized nutritional screening to evaluate its impact on PI prevention and healing, in line with existing methodological recommendations (12, 13).

This multicenter cohort study conducted in specialized rehabilitation centers provided detailed insights into the prevalence and characteristics of PIs among patients admitted to post-acute care. PIs were frequently present at admission, predominantly stage 1 and 2, and may have originated from previous hospital stays, highlighting the need for preventive strategies to be implemented early in acute care settings.

During rehabilitation, PIs significantly affected clinical management, functional recovery, and healthcare resources. Only a portion of lesions healed during the rehabilitation stay, underscoring the importance of ongoing prevention and targeted interventions.

Strengthening PI prevention in acute care, implementing structured management pathways in rehabilitation, and integrating comprehensive nutritional assessment and intervention are essential to improving patient outcomes, facilitating functional recovery, and optimizing healthcare resource utilization.

## Data Availability

The raw data supporting the conclusions of this article will be made available by the authors, without undue reservation.
