# Supplementary material for: Pressure injuries beyond acute care hospitals: a multicenter cohort study across rehabilitation settings
Source: Front Rehabil Sci. 2026 May 29;7:1808329. doi: 10.3389/fresc.2026.1808329 (PMC13260522; doi:10.3389/fresc.2026.1808329)
Supplement: Supplementary file 1 [file Datasheet1.docx]

| **Table S1. Study variables, definitions, and coding** | | | | | | |
| --- | --- | --- | --- | --- | --- | --- |
| **Variable** | **Source of variable** | **Operational definition** | **Category coding** | **Coding for multivariable analysis** | **Reference category** | **Use** |
| ***At admission*** |  |  |  |  |  |  |
| Gender | Medical record | Male / Female recorded in chart | M: male \| F: female | M: male \| F: female | M | Descriptive and multivariable analysis |
| Age (years) | Medical record | Age in years | Continuous (years) | ≤80 \| >80 | ≤80 | Descriptive and multivariable analysis |
| Patient origin | Medical record | Provenance | Public hospital \| Accredited private hospital \| Home \| Residential facility \| Other | - | - | Descriptive only |
| MDC | Medical record | Primary diagnosis classified by Major Diagnostic Category | 01: nervous \| 04: respiratory \| 05: cardiovascular \| 08: musculoskeletal \| Other/Not available | 01: nervous \| 04: respiratory \| 05: cardiovascular \| 08: musculoskeletal \| Other/Not available | 05: cardiovascular | Descriptive and multivariable analysis |
| Diabetes | Medical record | Presence of diabetes as a comorbidity | Yes \| No | - | - | Descriptive only |
| Cardiovascular diseases | Medical record | Presence of cardiovascular disease as a comorbidity | Yes \| No | - | - | Descriptive only |
| Chronic pulmonary diseases | Medical record | Presence of chronic respiratory disease as a comorbidity | Yes \| No | - | - | Descriptive only |
| Neurological diseases | Medical record | Presence of neurological disease as a comorbidity | Yes \| No | - | - | Descriptive only |
| Muscle hypertonia | Medical record | Clinical assessment of a pathological and involuntary increase in muscle tone characterized by stiffness and resistance to passive stretching | Yes \| No | - | - | Descriptive only |
| Dysphagia | Medical record | Assessment from speech therapist | Yes \| No | - | - | Descriptive only |
| Modified-consistency diet | Medical record | Modified consistency diet according to International Dysphagia Diet Standardisation Initiative | Yes \| No | - | - | Descriptive only |
| Feeding | Medical record | Ability to feed | Independent: no difficulty in feeding \| Partially assisted: requires some support or supervision to feed \| Fully assisted: unable to feed independently | Independent or partially assisted \| Fully assisted | Independent or partially assisted | Descriptive and multivariable analysis |
| Braden Score | Medical record | Score assessing the risk of developing pressure ulcers in bedridden patients or those with reduced mobility | ≥17: minimal risk \| 13–16: low risk \| 9–12: moderate risk \| ≤8: high risk | ≥17 \| <17 | ≥17 | Descriptive and multivariable analysis |
| Barthel Index | Medical record | 10-item ordinal scale used to measure functional independence in Activities of Daily Living (ADLs) | ≥61: mild dependence, 41–60: moderate dependence, 21–40: severe dependence, ≤20: total dependence | >40 \| ≤40 | >40 | Descriptive and multivariable analysis |
| Vascular access | Medical record | Type of vascular access | None \| Peripheral \| Central | - | - | Descriptive only |
| Incontinence | Medical record | Urinary or urinary and fecal incontinence | None \| Urinary only \| Urinary and fecal | - | - | Descriptive only |
| Urinary catheter | Medical record | Presence of an indwelling urinary catheter | Yes \| No | Yes \| No | No | Descriptive and multivariable analysis |
| Anti-decubitus devices | Medical record | Prescription of anti-decubitus devices | Yes \| No | - | - | Descriptive only |
| Mobilization prescription | Medical record | Prescription for mobilization | Yes \| No | - | - | Descriptive only |
| Restraints | Medical record | Use of physical restraints | Yes \| No | - | - | Descriptive only |
| ***During hospitalization*** |  |  |  |  |  |  |
| Cardiovascular/respiratory  event | Medical record | Occurrence of any cardiovascular or respiratory complication | Yes \| No | - | - | Descriptive only |
| Healthcare-acquired  infection | Medical record | Occurrence of any infection documented during hospitalization | Yes \| No | - | - | Descriptive only |
| Fall | Medical record | Occurrence of any fall during hospitalization | Yes \| No | - | - | Descriptive only |
| Mood alteration | Medical record | Occurrence of any mood or behavioral change during hospitalization | Yes \| No | - | - | Descriptive only |
| Type of discharge | Medical record | Discharge type | Routine \| Transfer \| Death \| Voluntary | - | - | Descriptive only |
| Length of stay (days) | Medical record | Total length of hospital stay (days) | Continuous (days) | - | - | Descriptive only |
| Onset of PI during  hospitalization | Medical record | Occurrence of ≥1 PIs after admission | Yes \| No | - | - | Descriptive only |
| PI = pressure injury |  |  |  |  |  |  |

| **Table S2.**  **Sensitivity analyses using cause-specific Cox regression models with robust standard errors clustered by facility** | | | | | | | |
| --- | --- | --- | --- | --- | --- | --- | --- |
| **Characteristic** | **HR** | **95% CI** | **P** |  | **aHR** | **95% CI** | **P** |
| At least 1 PI at admission *vs* none | 0.57 | 0.46–0.70 | <0.001 |  | 0.67 | 0.53–0.85 | <0.001 |
| Male *vs* female | 0.95 | 0.77–1.16 | 0.580 |  | 0.99 | 0.89–1.10 | 0.810 |
| Age > 80 years *vs* ≤80 years | 1.06 | 0.86–1.30 | 0.595 |  | 1.09 | 0.90–1.33 | 0.371 |
| MDC  01 *vs* 05  08 *vs* 05  04 *vs* 05  Other/Not Available *vs* 05 | 0.16  0.34  0.40  0.21 | 0.13–0.19  0.22–0.53  0.27–0.58  0.13–0.33 | <0.001  <0.001  <0.001  <0.001 |  | 0.22  0.40  0.47  0.27 | 0.15–0.31  0.26–0.62  0.30–0.74  0.16–0.45 | <0.001  <0.001  0.001  <0.001 |
| Barthel index ≤40 *vs* >40 | 0.45 | 0.31–0.66 | <0.001 |  | 0.82 | 0.58–1.15 | 0.247 |
| Braden score <17 *vs* ≥17 | 0.49 | 0.40–0.59 | <0.001 |  | 0.71 | 0.61–0.84 | <0.001 |
| Fully assisted feeding *vs* partially assisted/independent feeding | 0.29 | 0.20–0.42 | <0.001 |  | 0.63 | 0.46–0.87 | 0.005 |
| Presence of urinary catheter *vs* no catheter | 0.46 | 0.31–0.68 | <0.001 |  | 0.77 | 0.59–1.01 | 0.058 |
| aHR = adjusted hazard ratio; CI = confidence interval; HR = hazard ratio; PI = pressure injury.  MDC = Major Diagnostic Category  01 = Diseases and disorders of the nervous system  08 = Diseases and disorders of the musculoskeletal system and connective tissue  05 = Diseases and disorders of the cardiovascular system  04 = Diseases and disorders of the respiratory system | | | | | | | |
